# Current perspectives on RNA secondary structure probing

**DOI:** 10.1042/BST20140084

**Published:** 2014-08-11

**Authors:** Julia Kenyon, Liam Prestwood, Andrew Lever

**Affiliations:** * University of Cambridge Department of Medicine, Box 157 Level 5 Addenbrooke's Hospital, Hills Road, Cambridge, Cambridgeshire CB2 0QQ, U.K.

**Keywords:** nucleotide, RNA structure, selective 2′ hydroxy acylation analysed by primer extension (SHAPE), structural analysis

## Abstract

The range of roles played by structured RNAs in biological systems is vast. At the same time as we are learning more about the importance of RNA structure, recent advances in reagents, methods and technology mean that RNA secondary structural probing has become faster and more accurate. As a result, the capabilities of laboratories that already perform this type of structural analysis have increased greatly, and it has also become more widely accessible. The present review summarizes established and recently developed techniques. The information we can derive from secondary structural analysis is assessed, together with the areas in which we are likely to see exciting developments in the near future.

## Why study RNA secondary structures?

The chemical nature of RNA enables it to fold into complex three-dimensional structures. Such structures can be as stable and as intricate as those formed by proteins. As our knowledge of RNA biology and the importance of regulatory RNAs increases, it is becoming clear that RNA structure, and changes within it, regulate many cellular processes [1–3]. Just as our understanding of protein biology is greatly enhanced by visualizing the protein structures formed and their post-translational modifications and interactions with ligands, we need to examine RNA structures in order to comprehend their mechanisms of action. The RNAs of interest can be thousands of nucleotides in length, effectively precluding the use of X-ray crystallography or NMR. The secondary structures of such RNAs can however be studied and recent developments in biochemical probing techniques have greatly improved the speed and accuracy of such analyses.

## RNA secondary structural probing: basic methods and reagents

Many different compounds and enzymes are known to react with specific single-stranded ribonucleotides, or with double-stranded regions [4]. Until recently the conventional approach relied on using a combination of these to acquire a picture of the single- and double-stranded regions of the RNA. Using the standard gel electrophoresis methods to examine the reactivities (described below) this is a laborious process. In addition, it is difficult to build up complete coverage of the RNA, as most of these reagents react anomalously with some bases, or display a hierarchy of reactivity that is incompletely understood. For example, the enzyme CV1 cleaves only a subset of double-stranded nucleotides, with a preference for those in the middle of helices [5], and lead acetate cleaves most single-stranded nucleotides, but also some within helices [6]. Compounds or enzymes are used at a low concentration, so that, on average, there is less than one modification per transcript. Higher concentrations can give a false picture as the RNA may rearrange structurally following cleavage or modification.

After chemical or enzymatic probing, modification sites are sought. Commonly, RT (reverse transcriptase) is used to make a cDNA that terminates at the adduct or cleavage site. cDNAs are radioactively labelled and separated by gel electrophoresis alongside a sequencing ladder. Alternatively, if cleaving reagents are used, RNAs may be end-labelled and separated by electrophoresis without the reverse transcription step. Quantification of each cDNA or RNA product is by densitometric analysis. RNA secondary structures are then modelled using minimal free-energy prediction, using a software package such as Mfold [7] or RNA structure [8].

## SHAPE (selective 2′ hydroxy acylation analysed by primer extension)

In recent years, a group of compounds known as SHAPE reagents have become widely used for structural probing [9]. These compounds acylate the ribose in the sugar-phosphate backbone where it is flexible. This occurs in single-stranded rather than canonically base-paired regions. Acylation generates a bulky ester adduct that blocks the progress of RT. The reagents are powerful as they react with the backbone at every base, allowing rapid acquisition of comprehensive structural information. In effect most of the information about where an RNA is single- or double-stranded can be acquired in a single SHAPE probing experiment.

The power of the technique has been further enhanced by quantifying each of the cDNA products by labelling with fluorophores and analysing with capillary electrophoresis (‘high-throughput SHAPE’ [10]). To do this, the acylating reagent positive sample is labelled with one fluorophore and the negative control RNA-only sample is labelled with another. Two further fluorophores are used to make a sequencing ladder, either by reverse transcription, incorporating one ddNTP, or by cycle-sequencing. Three to four hundred nucleotides of data can be generated and read from each capillary, compared with 100–200 in a conventional gel. The ability to run 96 samples simultaneously further speeds up the process. Additionally, separation by capillary is less technically challenging than manual manipulation of large, radioactive sequencing gels, leading to an increase in the amount of readable data. Owing to the obvious benefits in speed, quality and amount of data acquired, as well as the avoidance of radioactivity, this fluorophore labelling and capillary electrophoresis technique has also been applied to the conventional biochemical and enzymatic probing reagents [11,12].

## How can we use SHAPE to generate the most structural information?

### Understanding low and high reactivity

SHAPE reagents acylate the 2′ hydroxy group of the ribose, reacting much more readily where the backbone is flexible, hence single-stranded, than where it is rigid, or Watson–Crick base paired. This is thought to be because unconstrained nucleotides more often display rare conformations that enhance the nucleophilicity of the 2′ hydroxy group [9]. During data fitting, each nucleotide is assigned a numerical reactivity value that represents how readily that nucleotide has been able to react with the SHAPE reagent, and the SHAPE dataset is normalized such that a value of approximately 0–0.3 is unreactive, and a value of approximately 0.7–1 or above is reactive [8,13]. Nucleotides that are Watson–Crick paired within helices usually lie within the numerical range that indicates unreactivity. However, not all double-stranded nucleotides display a low or even intermediate reactivity: the closing pairs of helices and adjacent nucleotides within the helix can be more prone to acylation and occasionally nucleotides that are canonically paired in the middle of helices can show a reactivity of 1 or more. This highlights the need to use flexible energetic penalties rather than hard constraints during the structural modelling process.

Many nucleotides that are within single-stranded regions display backbone flexibility and react with a normalized SHAPE value of approximately 0.7–1. However, approximately 2% of nucleotides within a structure exhibit very high SHAPE reactivity (>2) [14]. Many of these nucleotides, when examined *in crystallo*, are in dynamic regions, but in some, rather than being single-stranded and flexible, the backbone is thought to be constrained in a conformation that makes the 2′ hydroxy group hyper-reactive to SHAPE reagents. These fall into specific structural categories: the best characterized example is cAMP, which displays a reactivity of approximately 15 on the normalized scale of SHAPE reactivity [14]. The 2′ hydroxy group is thought to be so highly reactive because the non-bridging oxygen atoms of the 3′ phosphodiester bond are orientated away from it, which electrostatically stabilizes the transition state intermediate and makes the reaction more likely to proceed. This general ‘phosphodiester-away’ structure and mechanism accounts for one in seven hyper-reactive nucleotides. Another mechanism by which acylation readily occurs is through base-catalysis [14]. Here, a proximal functional group is present that is capable of forming a hydrogen bond with the 2′ hydroxy group, and the reaction proceeds via deprotonation and base catalysis. Within nucleotides that are highly reactive to SHAPE reagents, the C2′ endo conformation is greatly overrepresented; this is thought to allow the 2′ hydroxy group to interact favourably with nearby functional bases, such as purine N3 or pyrimidine O2 groups. However, the functional groups capable of performing this base catalysis can also be on non-adjacent nucleotides; thus the highly reactive nucleotides may give us an indication of through-space interactions in the RNA.

### Understanding intermediate reactivity

The definition of 0–0.3 as unreactive (paired) and 0.7 and above as reactive (unpaired) leaves a window of reactivity in between these values, containing a large proportion of the nucleotides, which are defined as neither unreactive nor reactive. Some of these nucleotides of intermediate reactivity are the closing pairs of helices, but many appear to be within single-stranded or internally paired regions. Deciphering the code of these nucleotide reactivities will lead to further advances in our modelling capabilities.

Since there are single-stranded conformations of the backbone that enhance the likelihood of acylation at the 2′ hydroxy group [14], there are likely to be conformations that diminish it. These have not yet been examined, but it is possible that they account for some of the single-stranded nucleotides of low to intermediate reactivity.

Another factor that influences nucleotide reactivity is the identity of the base: purines are approximately 1.5-fold more reactive than pyrimidines, with cytosine consistently displaying the lowest reactivities amongst unpaired nucleotides. These differences in reactivity between different bases that adopt similar structures are smaller than those between paired and unpaired nucleotides and hence were proposed to have minimal impact on structural prediction [15]. However, as our structural refinement capabilities increase, taking into account these differences could enhance the accuracy of our modelling. For example, a cytosine of intermediate reactivity is presumably more likely to be unpaired than is a guanosine of the same reactivity.

Intermediate reactivity can also result from the presence of a structural switch: in some cases, two or more stable structures can be present, with the differences in base pairing between them leading to differences in reactivity of certain nucleotides [16]. One clue to the existence of a structural mixture is the presence of intermediate to high reactivity on one side of a helix, with lower reactivities on the opposite side. In this situation, the unreactive strand could be paired (differently) in both structures, with the more reactive side of the helix remaining single-stranded in at least one structure within the mixture being probed. These different RNA conformers can have a similar electrophoretic migration and hence remain unidentified initially. Previous studies of the FIV (feline immunodeficiency virus) packaging signal RNA, using enzymes and conventional biochemical probing reagents, showed both single-strand- and double-strand-specific reagent cleavage within the same region. This was initially proposed to reflect the high G-U content of the helices, and hence their ‘breathability’ [5]. As SHAPE reagents probe the structure at every nucleotide, the reactivity differences on each side of the helices were much more readily seen, and the initial enzymatic probing results could then be used in the modelling of the two structures present. These experiments highlight not only the use of SHAPE in detecting the presence of structural switches, but also the usefulness of probing with other conventional enzymes and chemicals, alongside SHAPE reagents.

## Improving accuracy and reproducibility: using SHAPE to probe and model individual structures within a mixed population

For some RNAs whose secondary structure has been probed and modelled multiple times by different groups, both raw data and the ensuing structural predictions can be remarkably disparate [10,17–21]. This can be partially explained by the use of RNAs of differing lengths, and variable refolding and probing conditions. Although it is vital to examine *in vitro* an RNA that contains the motifs of physiological relevance, and to refold it and probe it under conditions that resemble its natural environment as closely as possible, many larger RNAs exist as a mixture of different structures *in vitro* (which may reflect the situation *in vivo*) [22]. These structures may be different conformers of the monomeric RNA, or they may be multimers [19]. In order to probe these structures individually, attempts were made in the past to stabilize individual conformers using non-physiological buffers, or by predicting the structures *in silico* or using data derived from a mixed structural population and introducing mutations that would hypothetically stabilize one conformer over the other [16,23].

We recently developed ‘in-gel SHAPE’ using SHAPE reagents to probe RNAs within a native acrylamide gel; this allows separation of conformers with differing electrophoretic mobilities and their individual analysis [24]. The technique allowed us to probe the structures of HIV-1 monomeric and dimeric packaging signal RNAs and to confirm that a previously proposed structural switch between the monomer and dimer does occur [21]. The technique was first validated using the well-characterized HIV-1 TAR (transactivation response element) stem–loop. Interestingly, probing the monomeric structure within the gel matrix was more reproducible than the standard conditions of probing in solution. The data also fit more closely the monomeric structure derived from crystallography and NMR experiments. On close examination faint bands that probably represent multimeric TAR RNAs were visible in the gel; cutting out and probing the structure of only the monomeric RNA excluded the signal generated by these structures [24]. In addition, we find that when we probe the same refolded RNA *in vitro* and in-gel, areas of high reactivity can be found in the *in vitro* probed sample that do not exist in any of the individual species visualized and in-gel probed [24]. This suggests that most *in vitro* refolding conditions result in a proportion of misfolded molecules whose electrophoretic migration differs too widely to form visible bands, or possibly that some form aggregates that are too large to migrate into the gel. These species may only make up a small proportion of the RNA; however, as some nucleotides are hyper-reactive to acylation by SHAPE reagents, with values up to 15 times the normal level of positive binding, they can have a significant effect on the overall SHAPE signal in some regions. In-gel probing may therefore be a more accurate way to structurally examine RNAs that form only one native, physiologically relevant structure, as well as to examine structural switches.

## Using SHAPE data to determine tertiary structures

As our understanding of the chemical behaviour of the SHAPE reagents with different RNA structural signatures increases, so too does the amount of data we can derive from an experiment. Different reagents have slightly different reactivity profiles and this can highlight the presence of different structures [25]. NMIA (*N*-methylisatoic anhydride) reacts slowly, and as a result will react to a greater extent than other SHAPE reagents with nucleotides undergoing structural changes over a long timescale. Its selective sites are often in the C2′endo conformation. 1M6 (1-methyl-6-nitroisatoic anhydride), on the other hand, favours an interaction with one face of the base, which can be exposed at the ends of some helices or in bulges or turns. Use of both NMIA and 1M6 in parallel experiments, known as ‘differential SHAPE’ can help to refine secondary structure and begin to shed light on tertiary structure [25]. Presumably, in the future, modelling programmes will factor in these data.

SHAPE can also be used in developing a tertiary structural model using other methods for which the secondary structural information must be known first. The technique is particularly useful as the reagents will react in a very wide range of buffers and conditions, so the secondary structure can be probed under exactly the same conditions as those used to probe the tertiary structure. This strategy was recently used to support modelling of the 3D structure of the HIV-1 packaging signal RNA using single-molecule FRET (fluorescence resonance energy transfer) [26], and has potential applications for SAXS (small-angle X-ray scattering) experiments. Three-dimensional data have also been generated using hydroxyl radical probing and capillary analysis, with a free-radical-generating moiety at a known location on the RNA [27].

## Higher-throughput methods

Recent developments in sequencing technology have now been applied to the RNA secondary structure probing pipeline. First, classical enzymatic probing strategies were applied to RNAs extracted from cells and analysed by next-generation sequencing [28–30]. In-cell DMS (dimethyl sulfate) probing has resulted in structural analysis of the transcriptome [31]. SHAPE technology has been applied to the deep sequencing of *in vitro* transcribed, barcoded RNAs [32]. The utility of such powerful genome-wide technology depends on how many RNAs are to be studied, and whether they are present in different spliced or polyadenylated forms. These variations might affect their structures and functions, but may not be accurately separated by next-generation sequencing, which tends to use fragmentation techniques to examine shorter segments of RNA. One recent development that could increase the accuracy of many different probing methods is a novel barcoding system to enable identification of overrepresented cDNAs [33]. This system also enables visualization of non-templated addition of nucleotides by the RT: such transcripts are then eliminated from the analysis.

## Examining low-abundance transcripts

The development of SHAPE reagents that can modify RNA inside living cells [34] means that the backbone flexibilities, and hence secondary structures of RNAs in the cytoplasm, can now be determined rapidly, using a single reagent. However, several picomoles of each RNA are required to examine the transcripts with capillary electrophoresis methods [10]. This makes high-throughput SHAPE useful for transcripts of high abundance, though unfortunately many RNAs of interest are minor species in the cytoplasm. The required sensitivity could be achieved using next-generation sequencing approaches, but it will not always be practical to use such powerful techniques to examine individual RNAs. However, a recently developed technique allows the amplification of signals from RNAs of low abundance [35]. This technique, alongside standard high-throughput SHAPE, means that structural analysis within cells should now be possible for all RNAs.

## Future perspectives

Secondary structure probing methods have advanced significantly in recent years, enabling rapid generation of structural data from most RNAs in diverse environments. There have been improvements in both the speed and accuracy of probing, in the number of reagents available to us and in the amount of structural information we can gain from them. Large datasets now enable us to evaluate evolutionary data on a new scale which is vitally important when considering whether a structure has been conserved and is hence likely to be functionally important. RNA functional elements within untranslated regions will often change in sequence but not in structure, a phenomenon widely observed in viral RNAs [5,36]. Modelling programmes are able to take much of this information into account, but not all at once, and users face a choice as to whether to model the lowest free-energy using a single set of SHAPE or other probing data or evolutionary conservation of structure. The recently developed SHAPEknots program [37] can also take into account unusual base-pairing forming a single pseudoknot, but once again only a limited amount of data can be interpreted, and there is no allowance for phylogenetic conservation of structure. Clues as to the tertiary structure, as well as our growing understanding of non-canonical interactions must also be considered. At present the different types of data must be interpreted separately and the structure modelled iteratively. Despite the drawbacks of having to use different packages and techniques during the modelling process, our understanding of RNA structure is at a very exciting stage.

## Abbreviations

1M6: 1-methyl-6-nitroisatoic anhydride
NMIA: *N*-methylisatoic anhydride
RT: reverse transcriptase
SHAPE: selective 2′ hydroxy acylation analysed by primer extension
TAR: transactivation response element
